# Age‐Dependent Remodeling of the Sciatic Nerve Proteome in 5xFAD Mice Can Be Attenuated by Exercise or Donepezil Treatment to Maintain Neuromuscular Function

**DOI:** 10.1111/acel.70595

**Published:** 2026-06-16

**Authors:** Matthew H. Brisendine, Dijanira Q. Nieves‐Esparcia, Orion S. Willoughby, Brieann Brown, John R. Brown, Daniel S. Braxton, Shelby N. Henry, Colin S. McCoin, John P. Thyfault, Jill K. Morris, Steven Poelzing, Robert W. Grange, Timothy J. Jarome, Charles P. Najt, Joshua C. Drake

**Affiliations:** ^1^ Department of Human Nutrition, Foods, and Exercise Virginia Polytechnic Institute and State University Blacksburg Virginia USA; ^2^ School of Neuroscience Virginia Polytechnic Institute and State University Blacksburg Virginia USA; ^3^ Department of Biological Sciences Virginia Polytechnic Institute and State University Blacksburg Virginia USA; ^4^ Department of Cellular Biology and Physiology and Department of Internal Medicine‐Division of Endocrinology University of Kansas Medical Center Kansas City Kansas USA; ^5^ Department of Neurology and KU Alzheimer's Disease Research Center University of Kansas Medical Center Kansas City Kansas USA; ^6^ Translational Biology, Medicine, and Health Program Virginia Polytechnic Institute and State University Roanoke Virginia USA; ^7^ School of Animal Sciences Virginia Polytechnic Institute and State University Blacksburg Virginia USA; ^8^ Metabolism Core Virginia Polytechnic Institute and State University Blacksburg Virginia USA

**Keywords:** 5xFAD, Alzheimer's disease, donepezil, exercise, nerve, neuromuscular, voluntary wheel running

## Abstract

Alzheimer's disease (AD) progresses along a continuum for years to possibly decades prior to cognitive decline. Although AD is primarily an age‐related brain pathology, increasing evidence indicates dysfunction in peripheral nerves and skeletal muscle may manifest early in the disease progression. However, the underlying cause(s) for peripheral nerve dysfunction leading to impaired skeletal muscle torque production are not understood. Sciatic nerves from 5xFAD and wild‐type (WT) mice were analyzed by tandem mass tag (TMT)‐labeled proteomics at 3, 4, and 7 months, identifying proteome remodeling coincides with functional declines at 4 months particularly in pathways linked to mitochondrial turnover, calcium handling, and inflammation. We hypothesized either voluntary wheel running or donepezil treatment, begun prior to neuromuscular decline, would delay manifestation of neuromuscular impairment in 5xFAD mice. Separate cohorts, using 3‐month‐old 5xFAD mice and WT littermates, were given voluntary wheel access for 4 weeks or treated with the acetylcholinesterase inhibitor donepezil. We assessed tibial nerve stimulated plantar flexion torque and sciatic nerve compound (motor) neuron action potential (CNAP) in vivo at 4 months. Both exercise and donepezil attenuated in vivo nerve‐stimulated muscle torque and CNAP dysfunction. Further, both exercise and donepezil attenuated the proteomic remodeling of the sciatic nerve through both shared and independent mechanisms that converged on mitochondria‐centric pathways. Our findings in the 5xFAD model of AD support the notion that early phenotypes of AD are evident in the periphery that may have implications for timing of interventions.

## Graphical abstract

Sciatic nerve proteome remodeling, particularly of mitochondrial‐centric pathways, occurs at 4 months of age in 5xFAD mice, coinciding with onset of neuromuscular dysfunction. Exercise or the acetylcholinesterase inhibitor donepezil, begun prior to onset, delays these phenotypes, suggesting early peripheral phenotypes of Alzheimer's Disease may have interventional implications. Created in Biorender.

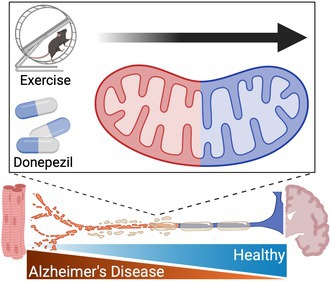

## 1 Background

The pathogenesis of Alzheimer's disease (AD) progresses along a continuum, with a preclinical phase that can span years to possibly decades before manifestation as mild cognitive impairment (MCI) and eventually clinical AD diagnosis (Sperling et al. 2011; Linn et al. 1995). A better understanding of phenotypes and related mechanisms of preclinical AD may bring to light novel early diagnostic strategies and interventions. Although AD is primarily an age‐related brain pathology, growing evidence indicates peripheral physiological changes, such as the deterioration of skeletal muscle mass and function that occur before noticeable cognitive decline, may serve as early indicators of AD during the preclinical period. Declines in muscle mass, strength, and innervating motor nerve conduction are linked to the severity of cognitive decline and development of MCI and AD (Qian et al. 2022; Buchman et al. 2007; Burns et al. 2010; Brenowitz et al. 2022). Alternatively, those who retain more muscle mass and strength are less likely to develop cognitive impairment or AD (Burns et al. 2010; Brenowitz et al. 2022; Soto et al. 2012; Ogawa et al. 2018; Kargazhanov et al. 2026; Zayia and Tadi 2023). Recently, in a cultured neuromuscular junction model derived from familial AD (fAD) containing induced pluripotent stem cells (iPSC), it was demonstrated that neuromuscular dysfunction still manifested (Kargazhanov et al. 2026), suggesting AD‐related neuromuscular dysfunction can occur in absence of cognitive deficits. Thus, a mechanistic understanding of early neuromuscular dysfunction in AD may improve our understanding of AD progression and offer novel avenues for disease‐modifying intervention.

Depolarization of α motor neurons trigger skeletal muscle contraction by transmitting action potentials along their axons to the neuromuscular junctions of innervated muscle fibers (Zayia and Tadi 2023). Declines in peripheral nerve conduction speed have been observed in MCI and AD subjects (Qian et al. 2022), thus extending the AD‐neurologic phenotype to the periphery. We recently showed the 5xFAD mouse model of AD develop neuromuscular dysfunction prior to the manifestation of cognitive impairment (Brisendine et al. 2024). The 5xFAD mouse is a well‐established model of AD that develop substantial extracellular amyloid‐beta plaques by 4 months of age, with cognitive decline becoming apparent shortly afterwards, though variance in the onset of observable cognitive decline has been reported (Forner et al. 2021; Oakley et al. 2006). We found 5xFAD mice develop impaired tibial nerve‐stimulated plantar flexion torque beginning at 4 months of age, as well as impaired sciatic nerve compound motor nerve action potential (CNAP) (Brisendine et al. 2024). Altered nerve‐to‐muscle communication in early AD may partly explain the inconsistent efficacy of interventional strategies, like exercise to slow disease progression (Morris et al. 2017; Greendale et al. 2021). However, what underlies peripheral nerve dysfunction in AD or related models, like 5xFAD, is unknown.

Donepezil is an FDA approved drug used to treat cognitive impairment in AD (Mehta et al. 2012; Guo et al. 2015; Rogers et al. 1998) that inhibits acetylcholinesterase, preventing breakdown of acetylcholine in the synaptic junction (Redman et al. 2022). In isolated skeletal muscle, donepezil increased tetanic torque production and endplate potential duration, suggesting treatment with donepezil may have peripheral benefits on neuromuscular function during early AD (Redman et al. 2022). This notion is supported by evidence that donepezil improves gait speed in individuals with mild AD (Montero‐Odasso et al. 2015). Additionally, MCI subjects taking donepezil had normal ADP‐stimulated mitochondrial respiration in skeletal muscle compared to impaired ADP‐stimulated respiration in MCI subjects not on donepezil (Morris et al. 2021).

The purpose of this study was to characterize temporal changes to the sciatic nerve proteome in 5xFAD mice as it relates to onset of indices of neuromuscular dysfunction (Brisendine et al. 2024) and test if exercise or donepezil could delay the initial decline in neuromuscular function seen at 4 months of age. Furthermore, we investigated whether attenuation of the initial decline coincided with maintenance in the sciatic proteome. We hypothesized that either voluntary wheel running or donepezil treatment would delay the manifestation of neuromuscular dysfunction in 5xFAD mice at 4 months of age. Understanding potential mechanism(s) behind neuromuscular dysfunction in a mouse model of AD and capacity to delay its manifestation may elucidate aspects of early AD identified in human subjects (Qian et al. 2022; Buchman et al. 2007; Brenowitz et al. 2022) and recapitulated in cultured systems (Kargazhanov et al. 2026) that could lead to new means to identify early signs of AD for intervention.

## 2 Methods

### 2.1 Animals

All experimental procedures were approved by the Institutional Animal Care and Use Committee (IACUC) at Virginia Tech. Male mice were housed in temperature‐controlled (21°C) quarters with a 12:12 h light–dark cycle and *ad libitum* access to water and chow (Purina). Heterozygous 5xFAD mice were obtained commercially (Jackson Laboratories, strain #034840). The 5xFAD mouse overexpresses five familial AD mutations [APP = Swedish (K670N, M671L), Florida (I716V), and London (V717I); PS1 = M146L and L286V] in forebrain neurons controlled by a *Thy1* promoter (Oakley et al. 2006). Male heterozygous 5xFAD mice and wild‐type littermates were obtained from heterozygous breeding pairs maintained in‐house. Genotype was confirmed via PCR.

### 2.2 Quantitative Proteomics of Sciatic Nerve

Whole sciatic nerve axon sections (~1 cm), including all the cell types therein, from both hind‐limbs were collected from five control (WT) and five 5xFAD mice at 3‐, 4‐, and 7‐months of age. For proteomics analysis, sciatic nerves were analyzed using the following protocol with minor modifications (Najt et al. 2023). Proteins were extracted with proteomic lysis buffer [PLB1: 7 M urea, 2 M thiourea, 0.4 M tris pH 8, 20% acetonitrile, 10 mM tris (2‐carboxyethyl) phosphine (TCEP), 40 mM chloroacetamide]. The samples were disrupted via tissue tearing and sonication. After centrifugation, the supernatants were collected to determine protein concentration via Bradford assay. 100 μg total protein were prepared from each sample and a pooled sample was created for bridging across Tanden Mass Tag (TMT) experiments. Samples were diluted fivefold with water and then trypsin digested using a 1:40 ratio of trypsin to total protein. Samples were incubated overnight for 16 h at 37°C. After incubation, samples were frozen at −80°C and died in a vacuum concentrator. Each sample was cleaned with a 1 cc Waters Oasis MCX cartridge (Waters Corporation, Milford, MA), and the eluate dried in vacuo. Samples were resuspended in water and peptide concentration determination by BCA assay. Equal peptide amounts for each sample were further cleaned and desalted on a C18 column. The digested samples were labeled with TMT16plex Isobaric Label Reagent (Thermo Scientific) in accordance with the manufacture's specifications. After TMT labeling, all the samples were multiplexed together into a new 1.5 mL microfuge tube. The TMT sample was dried down in vacuo. The samples were offline fractionated as described previously (Chaanine et al. 2021). Each fraction was desalted on a C18 stage tip and reconstituted for analysis. Samples were separated on an Easy nLC‐1000 UHPLC and analyzed by tandem mass spectra in a data‐dependent manner with a Thermo Fisher Oritap Eclipse mass spectrometer. In brief, we processed the MS peptide spectra using Sequest (Thermo Fisher Scientific, San Jose, CA, in ProteomeDiscoverer 2.2). The mouse (taxonID 10,090) Universal Proteome target protein sequence database was downloaded from UniProt (www.uniprot.org/) on August 8, 2024, and was merged with a common lab contaminant protein database (http://www.thegpm.org/cRAP/index.html). The digestion enzyme was trypsin; the fragment ion mass tolerance was 0.08 Da and the precursor tolerance was 15 ppm. We set the variable modifications for oxidation of methionine (+15.9949), pyroglutamic acid conversion from glutamine (17.0265), deamidation of asparagine (+0.9840), protein N‐terminal acetylation (+42.0106) and TMT 16plex (+229.1629) modification of lysine and peptide N‐terminus. We specified carbamidomethyl of cysteine as a fixed modification. We used the Percolator algorithm with a concatenated target‐decoy database approach to control the false discovery rate (https://doi.org/10.1038/nmeth1113). We reported protein lists with a 1% FDR threshold. Reporter ion intensities were adjusted by correction factors in all samples according to the algorithm described in i‐Tracker78 according to the TMTpro 16plex Lot Number WF324548 product data sheet from ThermoFisher Scientific. Normalization was performed iteratively (across samples and spectra) on intensities, as described in statistical analysis of Relative Labeled Mass Spectrometry Data from Complex Samples Using ANOVA.79 Pooled protein samples from all samples in the two TMT experiments were used for normalization, using two TMT channels for each experiment. Medians were used for averaging. Spectra data were log‐transformed, pruned of those matched to multiple proteins, and weighted by an adaptive intensity weighting algorithm. Differentially expressed proteins were determined by applying a permutation test with an unadjusted significance level *p* < 0.05 corrected by Benjamini–Hochberg. We set the hypothesis test method to *t*‐test, and we reported *p*‐values that were adjusted using the Benjamini‐Hochberg correction for false discovery rate.

### 2.3 Contextual Fear Conditioning

Prior to behavioral training, mice were handled for a total of 4 days to habituate to the researchers. Days 1 and 2 took place in the animal housing room and consisted of 5 min of handling per mouse. Days 3 and 4 involved a short transport period to the behavioral testing room followed by 5 min of handling per mouse. Mice then underwent contextual fear conditioning and open field testing, with the latter occurring 3 days after completion of the fear conditioning testing. Contextual fear conditioning was conducted as described previously (Swilley et al. 2023). Our protocol utilized CleverSys's Operant Chambers (CSI‐CHM‐FLR‐M‐32; 26 × 32 × 31 cm) housed inside sound‐attenuating cubicles with shocks being delivered via the CleverSys animal shocker controlled by CleverSys FreezeScan software and their CSI Intergrated Control 2 box in conjunction with their Power Control System (CSI‐P‐lll). For contextual fear conditioning training, mice were placed in the chamber for a total of 5 min with the first 3 min consisting of a baseline period. After this baseline, animals were presented with two‐foot shocks consisting of a 2 s 0.4 mA shock presentation with a 58 s inter‐trial interval. Afterwards, mice were returned to their homecage and the apparatus was cleaned with 70% ethanol before the next trial. The next day contextual fear conditioning testing occurred. Mice were returned to the same chamber for a total of 5 min with no foot shock presentations, with freezing behavior monitored and scored via the CleverSys FreezeScan software. Data is reported as the average percentage time spent freezing.

### 2.4 Shock Reactivity

To access for normal sensory processing, videos recorded during the fear conditioning training sessions were analyzed for shock reactivity to each of the two presented shocks as previously published (Turner et al. 2026; Jarome et al. 2010). Shock reactivity was defined as the latency (time) to the first instance of freezing behavior following each shock presentation. Data are reported as latency in seconds.

### 2.5 Open Field and Locomotion

For our open field and locomotion testing an open field arena with the dimensions of 40 cm × 40 cm × 30 cm was utilized. Before testing the arena was cleaned with 70% ethanol and clean beading was scattered across the floor. The mouse was then placed in the center and allowed to roam freely for 5 min before being returned to their homecage. Videos were manually scored for both open field and locomotive task. Videos were overlayed with a 4 × 4 grid with total time spent in the center boxes being measured as an anxiety output and total number of grid crossings being used as a locomotive measure. Data are reported as time spent in the center (s) and total number of grid crossings.

### 2.6 Voluntary Wheel Running

Heterozygous 5xFAD and WT littermates were randomly assigned to voluntary wheel running or sedentary groups at 3 months of age. Mice chosen for voluntary wheel running were individually housed in cages with running wheels. Sedentary mice were group housed in cages between 2 and 5 mice per cage without running wheels. Voluntary wheel running mice were given free access to a running wheel for the duration of the study (Brisendine et al. 2024). All mice were provided food and water *ad libitum*.

### 2.7 Exercise Capacity

After 4 weeks of access to voluntary wheel running (4 months of age), all mice (including the sedentary group) were acclimated for 10 min at 13 m/min at 0% incline for 3 consecutive days prior to testing endurance capacity, as previously described (Brisendine et al. 2024; Laker et al. 2017; Drake, Wilson, Cui, et al. 2021). For voluntary running mice, running wheels were locked the night before testing. On the day of testing, mice performed an exhaustive exercise test on a treadmill set to 5% incline. Mice ran at 5 m/min for 2 min, after which treadmill speed was increased by 5 m/min every 2 min, until exhaustion. Exhaustion was defined as refusal to run, despite prodding the mice with a brush at the back of the treadmill for 20 s.

### 2.8 Donepezil Treatment

Apple‐flavored donepezil and placebo treats were purchased from Bio‐serve (Flemington, NJ, USA). Donepezil treats were compounded with a dosage of 3 mg/kg, resulting in 0.075 mg of donepezil per treat, based on an average male mouse body weight of 25 g. Mice in the donepezil or placebo groups were individually housed and given treats daily at 17:00 h (2 h prior to dark cycle) for 4 weeks until sacrifice.

### 2.9 In Vivo Muscle Function

Torque‐frequency relationship of the plantar flexor muscles was assessed at 4 months of age, as previously described (Brisendine et al. 2024). Briefly, torque via plantar flexion was measured by the torque (mN) applied to the foot pedal, and multiplied by the length of the foot pedal (m). The units were set to mN × m. Isometric torque was determined with the foot at 90° to the tibia (neutral position) over a series of stimulations at frequencies: 1, 10, 30, 50, 65, 80, 100, 120, 150, 180, and 200 Hz via F‐E2 platinum‐tipped needle electrodes (Natus Manufacturing, Gort, Ireland) to deliver stimulations from the 701C stimulator (ASI, Aurora, ON, Canada) via tibial nerve depolarization. To do so, two electrodes were taped together ∼3 mm apart and inserted percutaneously, distal from the knee, parallel along the tibia. Data were plotted as torque normalized to the mass of the right hind limb plantar flexors, i.e., triceps surae (mN × m/mg). Relative torque data were calculated as each respective mouse value for each time point/baseline torque × 100%.

### 2.10 Sciatic Nerve Compound Motor Neuron Action Potential

The night prior to compound nerve action potential (CNAP) measures, all running wheels were locked, donepezil and placebo mice received their treat, and all mice were fasted with *ad libitum* water. Mice were anesthetized with 3% isoflurane, and a unilateral sciatic nerve was exposed for stimulation as previously described (Brisendine et al. 2024). CNAP duration, an index of conduction in nerve bundles (Bhatt et al. 2017) was determined by a stimulus electrode placed at distance greater than 3 mm from a bipolar sensing electrode in differential amplifier mode with electrode spacing at 2 mm (2 × 10^−3^ m). The common ground for the bipolar sensing electrode and stimulation electrode was placed in the foot of the mouse. Signal from the bipolar electrode was amplified by a Hugo Sachs Electonik D‐79232 amplifier and digitized by a Powerlab 4/35 at a sampling rate of 4 kHz. The left sciatic nerve was stimulated at a period of 1 s, 10 mV, and 1 ms monophasic pulse using a model 4100 isolated high‐powered stimulator (A‐M Systems, Sequim, WA, USA).

### 2.11 Blood Serum Biomarker Analysis

Blood was collected into serum separator tubes as previously described (Morris et al. 2021) and spun down at 3500 g to separate serum and cell debris. Serum was frozen at −80°C until further analysis. Neurofilament light (NfL) was measured on a Simoa HD‐X machine in serum using the Neurology 4‐Plex E kit (Quanterix). Samples were analyzed in duplicate with appropriate calibrators and controls included.

### 2.12 Tissue Collection

Following Sciatic Nerve CNAP measures, blood was drawn via cardiac puncture. After which mice were sacrificed via cervical dislocation and tissues were collected and snap frozen in LN_2_.

### 2.13 Western Blotting

SNAP frozen Hippocampi were cryo‐homogenized via mortar and pestle and prepared for SDS PAGE as previously described (Drake, Wilson, Laker, et al. 2021) membranes were probed for Amyloid beta using Biolegend Purified anti‐β‐Amyloid, 1–16 Antibody (# SIG‐39320).

### 2.14 Quantitative Proteomics of Sciatic Nerve From Exercise and Donepezil Treated Mice

Exercised and donepezil treated mice were generated as described above. Similar to the quantitative proteomics of Sciatic Nerve data described above, sciatic nerve samples from 4‐month‐old exercise and donepezil treated mice were collected. Proteins were extracted with proteomic lysis buffer [PLB1: 7 M urea, 2 M thiourea, 0.4 M tris pH 8, 20% acetonitrile, 10 mM tris (2‐carboxyethyl) phosphine (TCEP), 40 mM chloroacetamide]. The samples were disrupted via tissue tearing and sonication. After centrifugation, the supernatants were collected to determine protein concentration via Bradford assay. 100 μg was prepared from each sample and a pooled sample was created for bridging across TMT experiments. Samples were diluted five‐fold with water and then trypsin digested using a 1:40 ratio of trypsin to total protein. Samples were incubated overnight for 16 h at 37C. After incubation, samples were frozen at −80°C and dried in a vacuum concentrator. Each sample was cleaned with a 1 cc Waters Oasis MCX cartridge (Waters Corporation, Milford, MA), and the eluate dried in vacuo. Samples were resuspended in water and peptide concentration determined by BCA assay. Equal peptide amounts for each sample were desalted on a C18 column. The digested samples were labeled with TMT16plex Isobaric Label Reagent (Thermo Scientific) in accordance with the manufacturer's specifications. After TMT labeling, all the samples were multiplexed together into a new 1.5 mL microfuge tube. The TMT sample was dried down *in vacuo*. The samples were offline fractionated as described previously (Chaanine et al. 2021). Each fraction was desalted on a C18 stage tip and reconstituted for analysis. Data were collected as described above.

### 2.15 Heatmap and Pathway Analysis of Proteomics Data

Pathway analysis, hierarchical clustering, and heat map production were carried out as previously described (Najt et al. 2016; Shang et al. 2022; de Hoon et al. 2004; Ashburner et al. 2000; Mi et al. 2019; Gene Ontology Consortium 2021; Ge et al. 2020). The hierarchical clustering and heat maps were generated using Cluster 2.0 from the Eisen Laboratory modified by Michiel de Hoon (http://bonsai.hgc.jp/~mdehoon/software/cluster/). Java Tree Viewer was used to view and color the heat map. Identification of KEGG and GO pathways was determined via ShinyGO v0.82 (https://bioinformatics.sdstate.edu/go/) and cross‐referenced using PANTHER (https://pantherdb.org/).

### 2.16 Statistics

Data are presented as mean ± SEM and were analyzed and plotted via GraphPad Prism 9.5.1. Data were analyzed via Student's *t*‐test when one variable was present, two‐way ANOVA when two variables were present, or repeated measures two‐way ANOVA when two variables were measured in the same animals over time. In groups with significant standard deviation differences, data were log transformed to further detect significance among groups. Post hoc analyses were only performed when a significant interaction between a categorical and a quantitative variable was found, which are indicated in the figures where applicable. Statistical significance was established a priori as *p* < 0.05.

## 3 Results

### 3.1 Assessment of Whole Sciatic Nerve Axon Proteome at Different Ages in 5xFAD Mice

**FIGURE 1:**
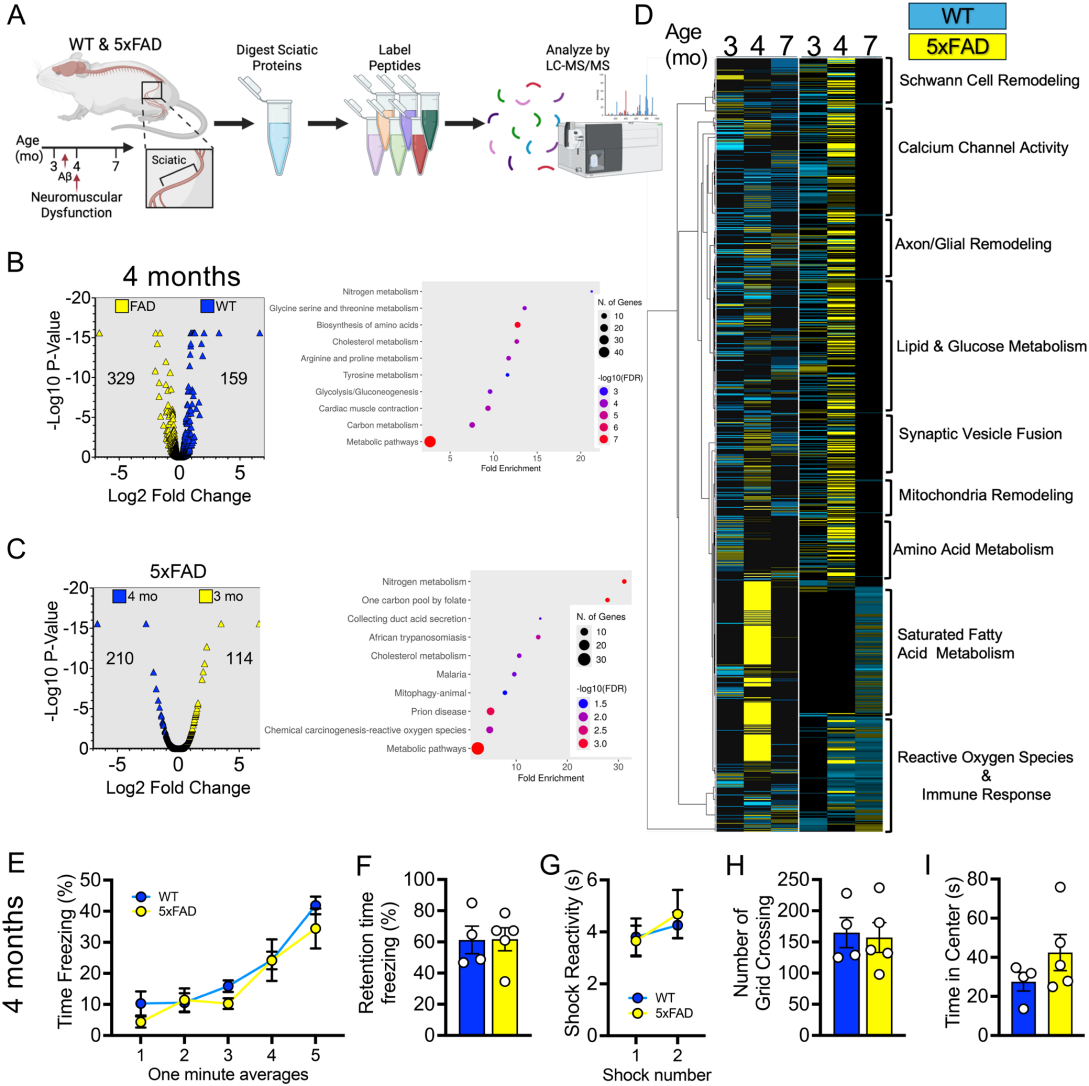
Cross sectional age time course assessment of sciatic nerve proteome in 5xFAD mice: (A) Schematic of the experimental design outlining proteomic profiling of sciatic nerve from 5xFAD mice at 3, 4, and 7‐months of age. (B) Volcano plot of 4‐month‐old WT and 5xFAD sciatic proteome (left) with corresponding KEGG pathway analysis of statistically significant proteins (right). Color indicators to designate genotype are square to avoid confusion with individual proteins represented by triangles. (C) Volcano plot of 3–4‐month‐old 5xFAD sciatic proteome (left) with corresponding KEGG pathway analysis of statistically significant proteins (right). Color indicators to designate genotype are square to avoid confusion with individual proteins represented by triangles. (D) Left: Hierarchical clustering of significantly different proteins between 3, 4, and 7‐month fractions (threshold for clustered proteins was determined by significance between groups). Right: Proteins were further grouped using *k*‐means statistics, breaking the 3, 4, 7‐month significant genes into 10 protein clusters. Blue = overexpressed in WT relative to 5xFAD at a given age; Yellow = overexpressed in 5xFAD relative to WT at a given age. (E, F) Contextual fear conditioning at 4 months of age. (G) Shock reactivity during contextual fear conditioning. (H) Grid crossing during open field. (I) Time spent in center. Data presented as mean ± SEM and two‐way ANOVA (E, G) or *t*‐test (F, H, I) was performed. *n* = 5 per age and genotype.

We previously demonstrated tibial nerve‐stimulated hindlimb skeletal muscle torque production declines at 4 months of age in 5xFAD mice due to impaired peripheral nerve function (Brisendine et al. 2024), analogous to findings in MCI and AD subjects as well as fAD iPSC‐derived motor neuron cultures (Qian et al. 2022; Brenowitz et al. 2022; Kargazhanov et al. 2026). Impairments in nerve transmission within the brain are known in 5xFAD mice (Kimura and Ohno 2009; Prince et al. 2021; Iaccarino et al. 2016) and in humans with AD (Terry et al. 1991; DeKosky and Scheff 1990; Selkoe 2002). However, to our knowledge, no analysis of the peripheral nerve proteome along the functional spectrum in an AD‐like model has been performed. To assess proteome changes over time in whole sciatic nerve axon of 5xFAD mice, we performed TMT‐labeled quantitative proteomic analysis on sciatic nerve axon sections from male 5xFAD mice and WT littermates at 3, 4, and 7 months of age (Figure 1A). Ages were chosen to represent when nerve‐to‐muscle communication is physiologically equivalent between groups (3 months), when neuromuscular dysfunction first appears (4 months), and at an advanced stage (7 months) (Brisendine et al. 2024). At 3 months, we observed 223 significantly abundant proteins upregulated in WT sciatic nerve axons compared to 72 in 5xFAD (Figure S1A). KEGG Pathway analysis of significantly enriched proteins revealed proteins were related to contraction and calcium signaling pathways (Figure S1A), although we previously observed equivalent nerve‐stimulated skeletal muscle torque production and sciatic nerve CNAP between 5xFAD and WT at 3 months of age (Brisendine et al. 2024). At 4 months of age, corresponding to the initial decline in nerve‐to‐muscle communication (Brisendine et al. 2024), the 5xFAD sciatic nerve axon proteome underwent a pronounced remodeling with 329 significantly abundant proteins enriched in the 5xFAD compared to 159 in WT (Figure 1B). KEGG Pathway analysis of significantly enriched proteins indicated nitrogen and amino acid metabolism were altered (Figure 1B), potentially alluding to changes in neurotransmitter regulation. At 7 months, the sciatic nerve axon proteome was again skewed towards WT with 143 abundant proteins enriched compared to 54 in 5xFAD (Figure S1B). KEGG Pathway analysis of significantly enriched proteins revealed proteins were related to fatty acid and amino acid metabolism (Figure S1B). As remodeling of the sciatic nerve axon proteome in 5xFAD mice mirrored previously observed decline in nerve‐stimulated muscle torque production between 3 and 4 months of age (Brisendine et al. 2024), we compared the 3‐ and 4‐month sciatic nerve axon proteomes in 5xFAD mice. The 5xFAD sciatic nerve axon proteome at 4 months of age had 210 abundant proteins compared to 114 at 3 months of age that related to amino acid and cholesterol metabolism, as well as mitophagy (Figure 1C), suggesting mitochondrial remodeling is a key event that coincides with neuromuscular dysfunction at 4 months of age in 5xFAD mice. To determine how specific sets of proteins in sciatic nerve axons were differentially enriched across age groups in 5xFAD mice compared to same aged WT littermates, enriched proteins were organized by hierarchical clustering and K‐means statistics (Figure 1D). Remodeling of the sciatic nerve axon proteome was most apparent in 5xFAD mice at 4 months of age with enrichments in proteins related to Schwann cell remodeling, calcium channel activity, axon remodeling, lipid/glucose metabolism, synaptic vesicle fusion, mitochondria remodeling, and amino acid metabolism (Figure 1D; middle row).

To determine the relationship between sciatic nerve axon proteome remodeling and cognitive state in 5xFAD mice at 4 months of age, we performed a battery of memory tests in a separate cohort of mice. WT and 5xFAD mice performed similarly during contextual fear conditioning (Figure 1E,F), which is a robust hippocampus‐dependent task that is widely used to test memory in rodents, suggesting no overt cognitive deficit at this age in our hands. Shock reactivity analysis (reaction to the foot shocks during training) showed no differences between groups (Figure 1G), suggesting no difference in sensory processing and, thus, supporting the contextual fear conditioning outcome. In the open field, 5xFAD and WT mice had a similar number of grid crossings (Figure 1H), suggesting that differences in locomotion/activity are not a factor in fear conditioning outcomes. Additionally, in open field testing, both WT and 5xFAD mice spent a similar amount of time in the center of the grid, suggesting no statistical differences in anxiety between genotypes at 4 months of age in our hands. Together, these data suggest that the onset of neuromuscular impairment at 4 months of age in 5xFAD mice (Brisendine et al. 2024) corresponds to sudden remodeling in the proteome of the sciatic nerve axon, particularly in pathways that converge on mitochondria that may precede the onset of overt cognitive decline. Thus, we hypothesized that the 3–4‐month age period in 5xFAD mice may represent an interventional window to maintain neuromuscular function.

### 3.2 Voluntary Wheel Running Attenuates Neuromuscular Impairment at 4 Months of Age in 5xFAD Mice

**FIGURE 2:**
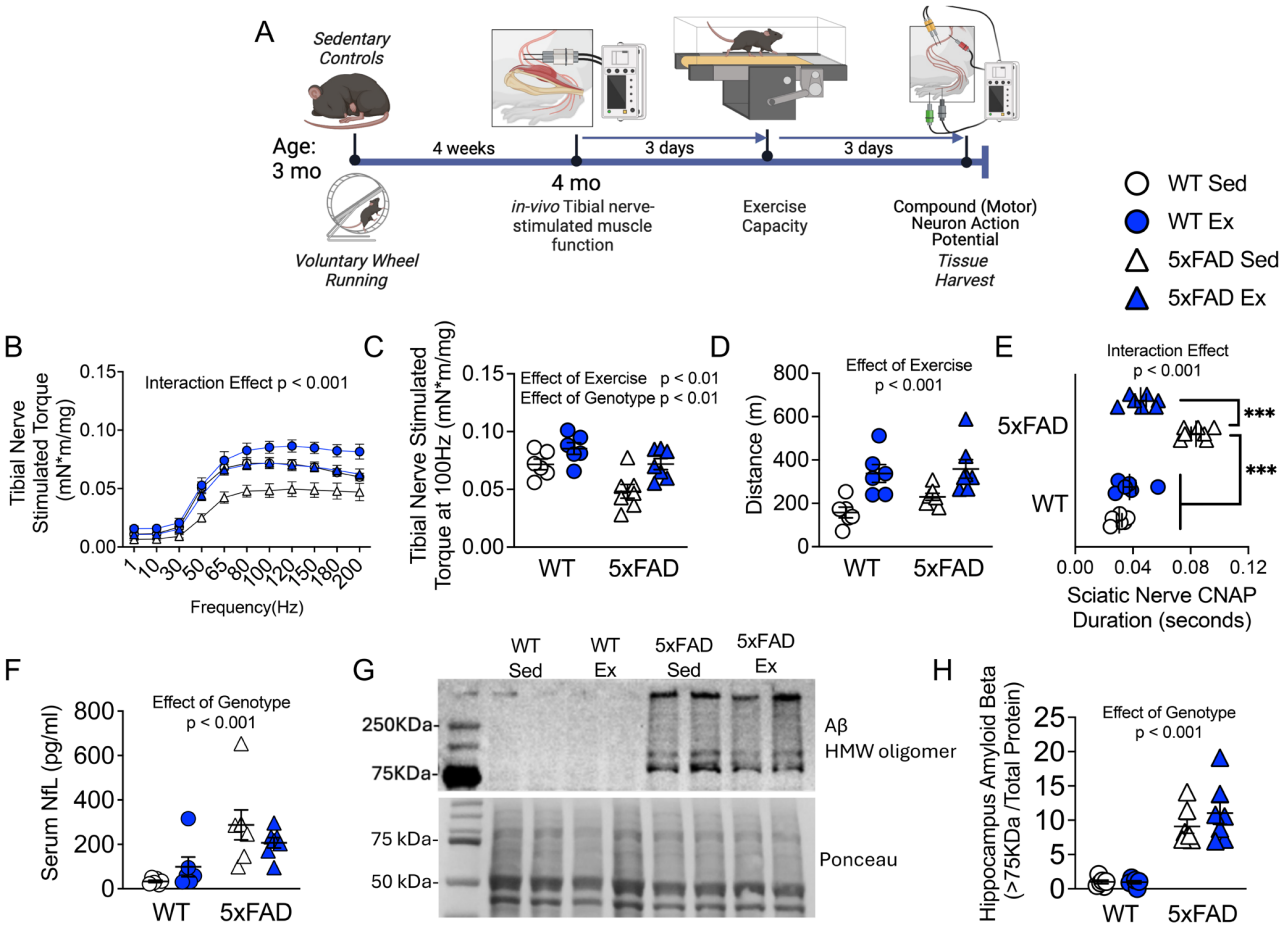
Voluntary wheel running attenuates neuromuscular impairment at 4 months of age in 5xFAD mice: (A) Schematic outlining study design of voluntary wheel running intervention. (B) Tibial nerve‐stimulated torque frequency of plantar flexors. (C) Tibial nerve stimulated torque at 100 Hz. (D) Exercise capacity. (E) In vivo compound nerve action potential (CNAP) of sciatic nerve. (F) Serum neurofilament light chain concentration in WT and 5xFAD. (G) High molecular weight A‐β oligomers in hippocampus. (H) Representative western blot and ponceau *n* = 6 per WT group and *n* = 6–7 per 5xFAD group. Data presented as mean ± SEM and repeated measures two‐way ANOVA was performed and Tukey's post hoc test performed when significant interaction between variables was observed (***p < 0.001). Panel A created in Biorender.

We have shown exercise training that went to 6 months of age in 5xFAD mice resulted in an altered adaptive response in skeletal muscle (Brisendine et al. 2024), suggesting a threshold where the pathological burden cannot be overcome by exercise. We hypothesized exercise begun prior to neuromuscular dysfunction may be able to delay onset of nerve‐stimulated skeletal muscle torque loss at 4 months of age in 5xFAD mice. 5xFAD mice and their WT littermates were given voluntary wheel running access for 4 weeks starting at 3 months of age (Figure 2A). To assess whether voluntary wheel running began at 3 months of age could prevent declines in skeletal muscle torque production we assessed torque production of the plantar flexors (i.e., gastrocnemius, plantaris, and soleus) via stimulation of the tibial nerve in vivo. Voluntary wheel running significantly improved plantar flexion torque production in WT, as evidenced by increase in the force frequency curve normalized to the wet weight of the triceps surae (Figure 2B). At 4 months of age, sedentary 5xFAD had a lower force frequency profile compared to WT, as we observed previously (Brisendine et al. 2024) (Figure 2B,C). Interestingly, exercise training maintained 5xFAD nerve‐stimulated torque frequency responses comparable to WT sedentary animals but there was no improvement beyond sedentary WT as seen in WT exercised mice (Figure 2B,C). Marginal differences in wet weights of the triceps surae muscles between groups did not reach statistical significance (Figure S2). Three days after measuring nerve‐stimulated skeletal muscle torque, exercise capacity was tested (Figure 2A). Exercise capacity significantly increased with voluntary wheel running in both WT and 5xFAD mice (Figure 2D), as reported previously (Brisendine et al. 2024). This outcome suggests that a lack of increased nerve‐stimulated skeletal muscle torque frequency between exercise WT and 5xFAD mice is not due to a difference in exercise performance capacity. To further examine the effect of voluntary wheel running on neuromuscular function, we assessed sciatic nerve CNAP in vivo 3 days following exercise capacity testing (Figure 2A). There was no effect of voluntary wheel running seen in WT Ex mice (Figure 2E), suggesting a possible functional ceiling in CNAP (Jones et al. 2022). Sedentary 5xFAD mice had significantly slower sciatic nerve CNAP compared to WT (Figure 2E), analogous to our previous findings (Brisendine et al. 2024). However, exercise training mitigated the increase in sciatic nerve CNAP in 5xFAD at 4 months of age (Figure 2E). Taken together these findings demonstrate 4 weeks of voluntary exercise can attenuate the initial decline in neuromuscular function at 4 months of age in 5xFAD mice.

**FIGURE 3:**
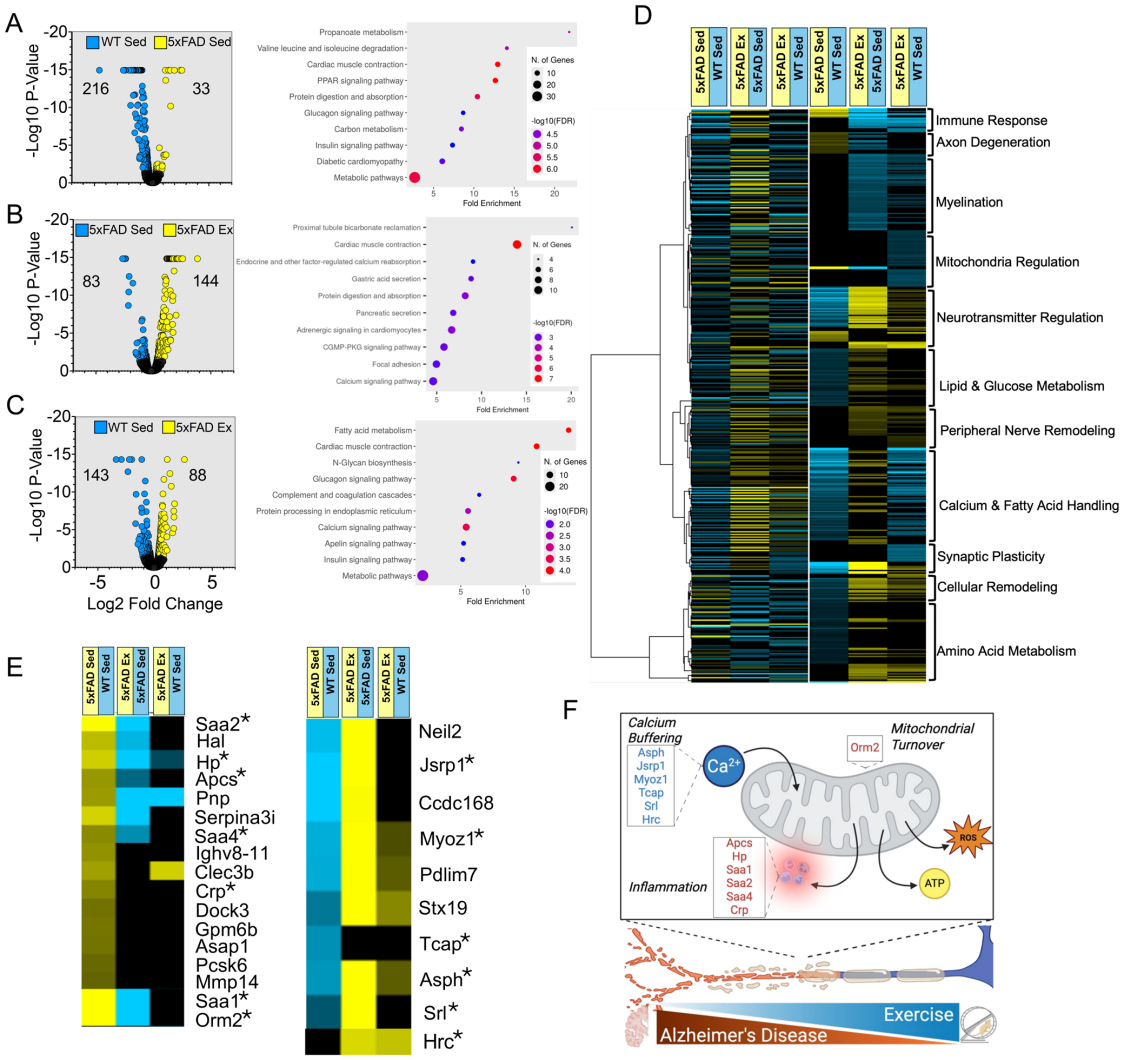
Proteomic profiling of sciatic nerve following exercise training: (A) Volcano plot of WT Sed and 5xFAD Sed sciatic proteome (left) and KEGG pathway analysis of statistically significant enriched proteins (right). (B) Volcano plot of 5xFAD Sed and 5xFAD Ex sciatic proteome (left) with corresponding KEGG pathway analysis of statistically significant enriched proteins (right). (C) Volcano plot of WT Sed and 5xFAD Ex sciatic proteome with corresponding KEGG pathway analysis of statistically significant enriched proteins (right). (D) Heatmap and K‐means statistical analysis of proteomes from WT and 5xFAD sciatic nerves. Left: Hierarchical clustering of proteins significantly different between WT and 5xFAD Sed and Ex (threshold for clustered proteins was determined by significance between groups). Right: Proteins were further grouped using k‐means statistics, breaking the significant genes into 10 protein clusters. Colors represent genotype the proteins were significantly enriched in (blue = WT, yellow = 5xFAD). (E) Heat map of proteins inversely affected by exercise and returned to WT levels. *Proteins that group together in STRING analysis. (F) Schematic of protein–protein interacting clusters, representing processes maintained to WT Sed by exercise in 5xFAD.

We next assessed if maintained nerve‐stimulated torque production and sciatic nerve CNAP in 5xFAD mice following 4 weeks voluntary exercise training at 4 months of age coincided with improved indices of AD‐like neuropathology. Circulating NfL chain in serum was significantly elevated in both sedentary and exercised 5xFAD mice compared to WT counterparts (Figure 3F). Expression of high molecular weight Aβ oligomers in hippocampus lysates was also elevated in both sedentary and exercise trained 5xFAD mice (Figure 3G,H). Together, these data suggest that attenuation of neuromuscular dysfunction in 5xFAD mice can result from exercise training independent of improvement in markers associated with the AD‐like phenotype of the model.

To determine if 4 weeks voluntary exercise attenuated the onset of neuromuscular decline in 4‐month‐old 5xFAD mice through mitigation of pathways notably undergoing remodeling at this age (Figure 1D), we performed TMT‐labeled proteomics of sciatic nerve axon sections in exercise trained mice and compared to sedentary cohorts. Following LC–MS/MS analysis of sciatic nerve axons, we identified 7982 proteins from a total of 18,636 unique peptide spectra at a 2‐peptide and a 1.1% FDR minimum. Of these 7982 proteins, 33 were significantly enriched in 5xFAD‐Sed compared to 216 in WT‐Sed, related to propanoate and amino acid metabolism, as well as PPAR signaling (Figure 3A). In 5xFAD exercised sciatic nerve axons, 144 proteins were enriched compared to 83 in sedentary counterparts, related primarily to calcium and contraction signaling (Figure 3B). Because exercise training maintained nerve‐stimulated torque frequency and sciatic nerve CNAP comparable to WT sedentary animals (WT‐Sed) (Figure 2B,C,E), we compared 5xFAD exercise sciatic nerve axon proteome to WT‐Sed. In 5xFAD exercised mice, 88 proteins were enriched in sciatic nerve axons compared to 143 in WT‐Sed related to fatty acid metabolism and calcium signaling (Figure 3C). Further analysis via hierarchical and K‐means clustering showed distinct differences between voluntary wheel running and genotype (Figure 3D, Figure S3A). Next, we focused on proteins whose expression was different between WT and 5xFAD sedentary animals, inversely affected by exercise in 5xFAD mice compared to sedentary, and then roughly equivalent with WT sedentary following exercise training in 5xFAD. A protein–protein interaction string map was generated from identified proteins (Figure S4B) and interacting clusters were identified (Figure 3E, Figure S2B). Identified protein–protein interaction clusters were related to inflammation (Apcs, Hp, Saa1, Saa2, Saa4, Crp), calcium buffering (Asph, Jsrp1, Myoz1, Tcap, Srl, Hrc), and mitochondrial turnover (Orm2) (Figure 3E,F and Figure S4B). These data suggest that exercise, when begun prior to onset of nerve‐stimulated torque loss and slowed sciatic nerve CNAP, attenuates neuromuscular dysfunction in 5xFAD mice by suppressing onset of proteome changes to inflammatory and calcium buffering processes that coincide with alterations in proteins related to mitochondrial turnover at 4 months of age (Figures 1D and 3F).

### 3.3 Donepezil Attenuates Neuromuscular Impairment at 4 Months of Age in 5xFAD Mice

**FIGURE 4:**
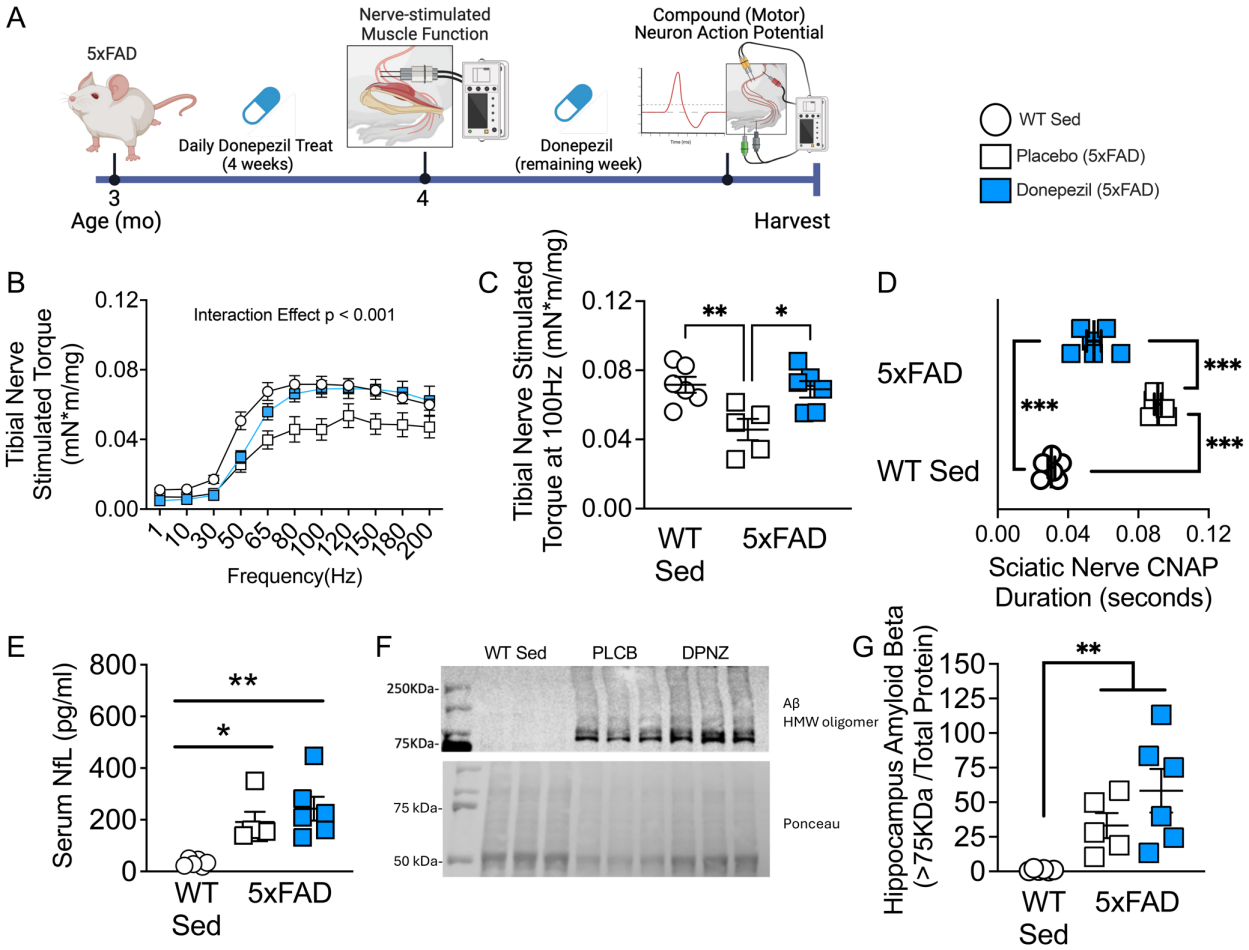
Donepezil attenuates neuromuscular impairment at 4 months of age in 5xFAD mice: (A) Study design of donepezil treatment. (B) Tibial nerve‐stimulated skeletal muscle torque frequency. (C) Tibial nerve stimulated torque at 100 Hz. (D) In vivo compound nerve action potential (CNAP) of sciatic. (E) Serum neurofilament light chain concentration in WT and 5xFAD. (F) High molecular weight A‐β oligomers in hippocampus. (G) Representative western blot and ponceau. *n* = 6 for WT, *n* = 6 for donepezil (DPNZ) and *n* = 5 for placebo (PLCB). Data presented as mean ± SEM and repeated measures two‐way ANOVA (B), two‐way ANOVA (C), or one‐way ANOVA (D, E, G) was performed and Tukey's post hoc test performed when significant interaction between variables was observed (*p < 0.05, **p < 0.01, and ***p < 0.001). Panel A created in Biorender.

As the efficacy of exercise as an interventional strategy in AD is dependent on a variety of factors, such as timing along the continuum of the pathology (Brisendine et al. 2024; Morris et al. 2017; Brisendine and Drake 2023), we tested whether a currently approved therapeutic may also delay neuromuscular decline in 5xFAD mice. The acetylcholinesterase inhibitor Donepezil is FDA approved to slow cognitive impairment in AD (Mehta et al. 2012; Guo et al. 2015). Donepezil has been shown to maintain mitochondrial respiration in skeletal muscle in humans with MCI (Morris et al. 2021) as well as gait speed (Montero‐Odasso et al. 2015). As sciatic nerve axon proteome changes at 4 months of age in 5xFAD mice involve mitochondria‐centric processes (e.g., mitochondrial turnover, calcium buffering, inflammation, and metabolism) (Figures 1D and 3D–F), we hypothesized treatment with donepezil could delay onset of neuromuscular dysfunction. Following 4 weeks of daily donepezil treatment, beginning at 3 months of age, we assessed in vivo tibial nerve‐stimulated plantar flexor torque production (Figure 4A). Donepezil prevented decline of plantar flexion torque production in 4‐month‐old 5xFAD mice compared to placebo (PLCB) treated mice when normalized to the wet weight of the triceps surae (Figure 4B,C). No differences in wet weights of the triceps surae muscles were found (Figure S2). Decline in sciatic nerve CNAP in vivo was attenuated in 5xFAD mice treated with donepezil compared to placebo but was still significantly slower compared to WT sedentary animals (Figure 4D). In sum, 4 weeks of donepezil treatment begun before neuromuscular dysfunction in 5xFAD mice can slow its development.

We also assessed if maintained nerve‐stimulated torque production and sciatic nerve CNAP in 5xFAD mice treated with donepezil for 4 weeks coincided with improvements in indices of AD‐like neuropathology. Serum NfL was significantly elevated in both placebo and donepezil treated groups compared to WT sedentary (Figure 4E). Expression of high molecular weight Aβ oligomers in hippocampus lysates was also elevated in both placebo and donepezil treated 5xFAD mice (Figure 4F,G). Together, these data suggest attenuated neuromuscular decline in 5xFAD mice can be obtained with donepezil treatment independent of improvement in markers associated with the AD‐like phenotype of the model.

**FIGURE 5:**
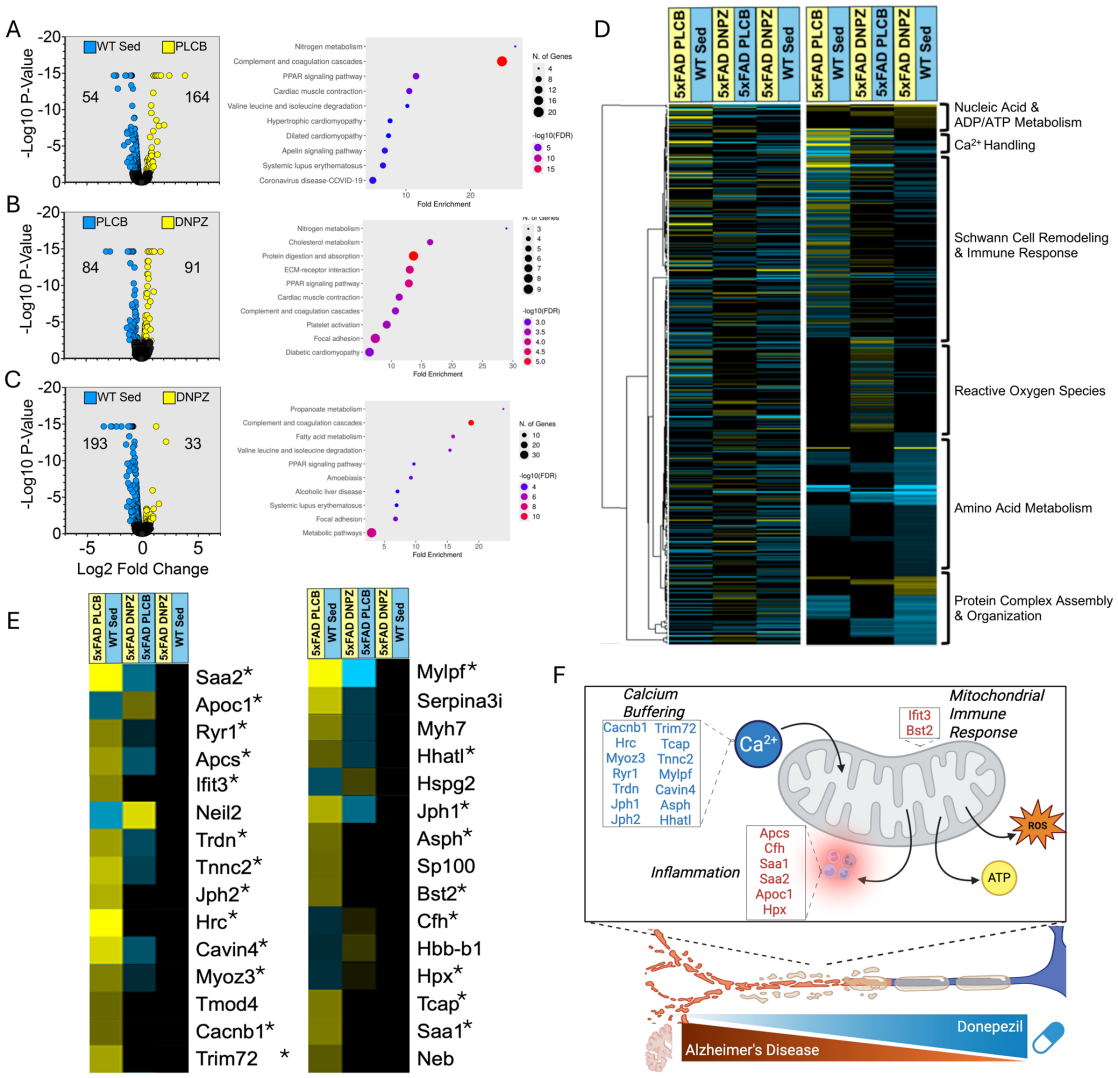
Proteomic profiling of sciatic nerve following donepezil treatment: (A) Volcano plot of WT Sed and 5xFAD placebo (PLCB) sciatic proteome (left) with corresponding KEGG pathway analysis of statistically significant enriched proteins (right). (B) Volcano plot of 5xFAD PLCB and 5xFAD donepezil (DNZP) sciatic proteome (left) with corresponding KEGG pathway analysis of statistically significant enriched proteins (right). (C) Volcano plot of WT Sed and 5xFAD DNZP sciatic proteome (left) with corresponding KEGG pathway analysis of statistically significant enriched proteins (right). Colors represent genotype the proteins were significantly enriched in (blue = WT, yellow = 5xFAD). (D) Heatmap and K‐means statistical analysis of proteomes from WT and 5xFAD sciatic nerves. Left: Hierarchical clustering of proteins significantly different between WT and 5xFAD PLCB and donepezil fractions (threshold for clustered proteins was determined by significance between groups). Right: Proteins were further grouped using k‐means statistics, breaking the significant genes into 10 protein clusters. (E) Heat map of proteins inversely affected by donepezil and returned to WT levels. *Proteins that group together in STRING clusters. (F) Schematic of protein–protein interacting clusters, representing processes maintained to WT Sed by donepezil in 5xFAD.

To determine if 4 weeks donepezil treatment attenuated the onset of neuromuscular decline in 4‐month‐old 5xFAD mice through mitigation of pathways notably undergoing remodeling at this age (Figure 1D), we again performed TMT‐labeled proteomics of sciatic nerve axon sections. Following LC–MS/MS analysis of sciatic nerve axons from donepezil treatment, we identified 5861 proteins from a total of 61,925 unique peptide spectra at a 2‐peptide and a 0.5% FDR minimum. Of these 5861 proteins, 164 were significantly enriched in 5xFAD‐PLCB compared to 54 in WT‐Sed (Figure 5A), related to nitrogen metabolism, amino acid metabolism, contraction, and PPAR signaling pathways, which overlaps with equivalent comparisons between 4‐month‐old 5xFAD and WT (Figure 1B) as well as 5xFAD sedentary mice (Figure 3A). In 5xFAD treated with donepezil, 91 proteins were enriched compared to 84 in placebo treated, related to nitrogen metabolism, protein metabolism, contraction and PPAR signaling pathways (Figure 5B). In 5xFAD treated with donepezil, 33 proteins were enriched compared to 193 in WT‐Sed, related to fatty acid metabolism, complement and coagulation cascades (inflammation), and metabolic pathways (Figure 5C), which were also observed in exercise trained 5xFAD mice (Figure 3C). Heatmaps of hierarchical and K‐means clustering showed distinct differences between 5xFAD‐DPNZ and PLCB in relation to WT‐Sed (Figure 5D). As donepezil treatment maintained neuromuscular function similar to WT‐Sed mice (Figure 4B–D), we again focused on proteins whose expression was different between WT and 5xFAD placebo treated animals, inversely affected by donepezil in 5xFAD mice compared to placebo, and then largely equivalent with WT sedentary following donepezil treatment in 5xFAD. Protein–protein interaction string map was generated from identified proteins (Figure S5) and interacting clusters were identified (Figure 5E and Figure S5). Identified protein–protein interaction clusters were related to mitochondrial immune response (Ifit3, Bst2), inflammation (Apcs, Cfn, Saa1, Saa2, Apoc1, Hpx), and calcium buffering (Cacnb1, Hrc, Myoz3, Ryr1, Trdn, Jph1, Jph2, Trim72, Tcap, Tnnc2, Mylpf, Cavin4, Asph, Hhatl) (Figure 5E,F; Figure S5). These data suggest that donepezil, when begun prior to onset of nerve‐stimulated torque loss and slowed sciatic nerve CNAP, attenuates neuromuscular decline in 5xFAD mice by suppressing onset of changes to mitochondrial immune and inflammatory, as well as calcium buffering processes (Figures 1D and 5F).

We next tested whether donepezil treatment could rescue neuromuscular function once manifested. We treated 5‐month‐old 5xFAD mice with donepezil or placebo for 4 weeks (Figure S6A), an age at which neuromuscular dysfunction is already present (Brisendine et al. 2024) and past the initial proteome remodeling at 4 months of age (Figure 1D), Following 4 weeks of treatment, at 6 months of age, we assessed tibial nerve‐stimulated plantar flexor torque production in vivo and direct muscle stimulation (Figure S6A). As we observed previously (Brisendine et al. 2024), nerve‐stimulated skeletal muscle torque was significantly lower compared to direct muscle‐stimulated torque production, again reiterating that the source of neuromuscular dysfunction was localized to the peripheral motor nerve and not to the skeletal muscle's capacity to produce torque during contraction (Figure S6B) (Brisendine et al. 2024). Compared to placebo treated animals, donepezil did not improve tibial nerve‐stimulated skeletal muscle torque production at 6 months of age (Figure S6C). In fact, at 6 months of age, donepezil treated animals had lower direct muscle‐stimulated torque production compared to placebo (Figure S6D), suggesting donepezil may have AD‐like disease stage‐dependent negative effects. Furthermore, in vivo sciatic nerve CNAP was not improved in 6‐month‐old 5xFAD mice treated with donepezil compared to placebo (Figure S6E). While donepezil treatment attenuates the initial loss of nerve‐stimulated muscle torque production and sciatic CNAP in 5xFAD mice at 4 months of age (Figure 4), treatment with the same dose cannot rescue neuromuscular function once manifested.

### 3.4 Shared and Unique Proteomic Profiles of the Sciatic Nerve in Voluntary Wheel Running and Donepezil

**FIGURE 6:**
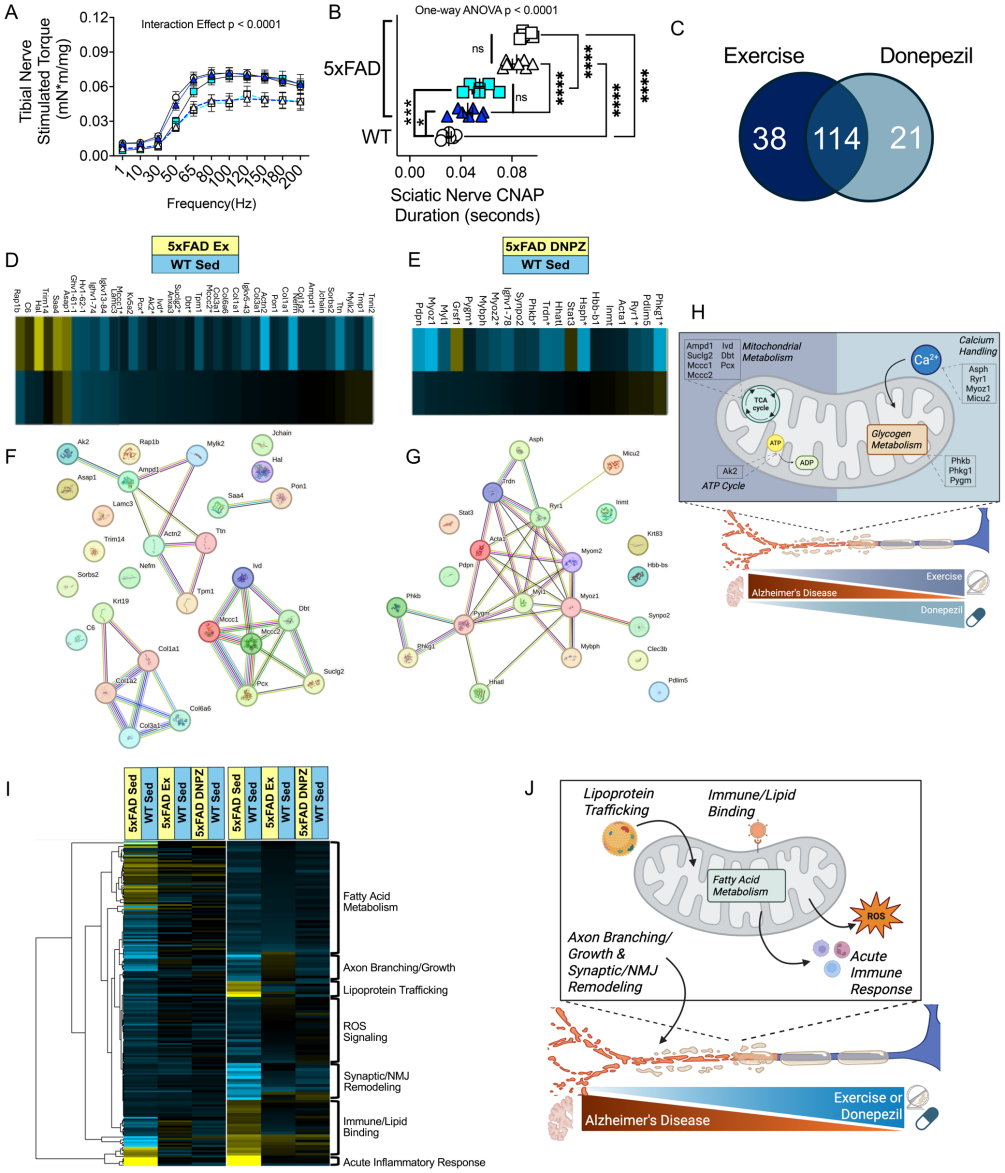
Shared proteomic changes in the sciatic nerve between exercise training and donepezil treatment: (A) Tibial nerve‐stimulated skeletal muscle function in donepezil (DNZP) and voluntary wheel running (Ex). (B) CNAP in donepezil and voluntary wheel running. (C) Venn diagram of significant shared and unique proteins identified in the proteomic screen with voluntary wheel running and DPNZ treatment. (D) Heatmap of unique proteins from 5xFAD Ex or (E) DNZP treated maintained relative to WT Sed. *Proteins that group together in STRING clusters. (F, G) STRING network was generated (https://string‐db.org) to examine distinct protein–protein interaction patterns among significantly enriched proteins following exercise training (F) or donepezil treatment (G). Nodes represent shared or treatment‐specific proteins, with edge thickness indicating interaction confidence. Clusters identify biological processes commonly or uniquely influenced by each intervention. (H) Schematic of unique processes maintained to WT Sed levels by both Ex and DPNZ in 5xFAD. (I) Left: Hierarchical clustering of shared proteins significantly different from WT and 5xFAD sedentary comparison following either Ex or DNPZ (threshold for clustered proteins was determined by significance between groups). Right: Proteins were further grouped using k‐means statistics, breaking the significant genes into 10 protein clusters. (J) Schematic of shared processes maintained to WT Sed levels by both Ex and DPNZ in 5xFAD.

Both exercise training and donepezil treatment in 5xFAD mice resulted in similar physiological maintenance of neuromuscular function as WT‐Sed mice when begun at 3 months of age (Figure 6A) and delayed increased CNAP (Figure 6B). Therefore, we sought to identify both shared and unique proteomic pathways through which exercise and donepezil were protective. For this comparison, we analyzed all sciatic nerve axon proteins in 5xFAD exercise and donepezil treated groups that were not significantly different from WT‐Sed, which resulted in 173 proteins that were attenuated to WT sedentary levels. We identified 114 proteins in common between 5xFAD exercise and donepezil groups (Figure 6C). 5xFAD exercise mice had 38 unique proteins that were not significantly different from WT‐Sed, whereas 5xFAD donepezil treated mice had 21 unique proteins (Figure 6C). Heatmaps of hierarchical and K‐means clustering were generated of the 38 proteins unique to exercise and 21 proteins unique to donepezil treatment (Figure 6D,E). Protein–protein interaction string maps were generated for proteins unique to exercise or donepezil to identify interacting clusters (Figure 6F,G). Protein–protein clusters identified unique to exercise training were related to TCA cycle (Ampd1, Suclg2, Mccc1‐2, Ivd, Dbt, Pcx) and ATP cycling (Ak2) (Figure 6F,H). Identified protein–protein interaction clusters in 5xFAD mice treated with donepezil that were uniquely maintained relative to WT Sed levels were related to calcium handling (Asph, Ryr1, Myoz1, Micu2) and glycogen metabolism (Phkb, Phkg1, Pygm) (Figure 6G,H). We next generated heatmaps of hierarchical and K‐means clustering of the 114 shared proteins between exercise training and donepezil treatment (Figure 6I), to elucidate shared biological processes between the two interventions that contribute to attenuation of the initial loss of nerve‐stimulated muscle torque production and sciatic nerve CNAP in 5xFAD mice at 4‐months of age. The 114 shared proteins between exercise and donepezil were involved in pathways related to trafficking and metabolism of fatty acids, synaptic transmission, and acute inflammatory response (Figure 6I,J). Thus, these data suggest exercise training and donepezil act, in part, by attenuating proteome changes related to diverse mitochondrial‐centric processes, suggesting onset of neuromuscular dysfunction at 4 months of age in 5xFAD mice may be due to proteomic changes to mitochondria.

## 4 Discussion

Neuromuscular impairments in preclinical AD are linked with risk and severity of cognitive decline (Buchman et al. 2007; Burns et al. 2010; Brenowitz et al. 2022; Soto et al. 2012; Ogawa et al. 2018; Boyle et al. 2009; Aggarwal et al. 2006) and recent evidence in fAD iPSC‐derived motor neurons suggests AD‐associated neuromuscular impairment can occur independent of cognitive deficits (Kargazhanov et al. 2026). However, little is understood of the potential mechanistic cause(s) of neuromuscular dysfunction in an AD context and whether its onset can be delayed. Previously, we discovered a decline in nerve‐stimulated muscle torque production and sciatic nerve CNAP in 5xFAD mice, a model of AD, that manifested at 4 months of age (Brisendine et al. 2024). In this study, we investigated the proteomic profiles of whole sciatic nerve axons from 5xFAD mice at 3, 4, and 7 months of age. Additionally, we investigated whether exercise training via voluntary wheel running or donepezil treatment initiated at 3 months of age can mitigate declines in neuromuscular function and alterations to the sciatic proteome that manifest at 4 months of age. We found distinct changes in the sciatic nerve axon proteome between WT and 5xFAD with significantly abundant proteins in several pathways, including mitochondrial quality control, immune response, metabolism, calcium regulation, and synaptic transmission at 4 months of age. Impairments in nerve‐stimulated muscle torque production, sciatic nerve CNAP, and the associated remodeling of the sciatic nerve axon proteome at 4 months of age occurred at an age that we were unable to show evidence of overt cognitive deficits, suggesting neuromuscular dysfunction is an early characteristic of the AD‐like pathology in 5xFAD mice. As motor deficits, including slowed peripheral nerve conduction, have been reported in MCI and AD subjects (Qian et al. 2022; Buchman and Bennett 2011) and are recapitulated in fAD iPSC‐derived motor neuron culture systems (Kargazhanov et al. 2026), our findings support the notion that early phenotypes of AD are evident in the periphery that may have interventional implications (Taha et al. 2025; Matthews et al. 2023; Cheng et al. 2020; Johnson et al. 2023; Lysaker et al. 2026; Kemna et al. 2025).

Whole proteomic changes in whole sciatic nerve axons of 5xFAD mice at 4 months of age share molecular profiles with established AD brain pathology, including alterations in several mitochondrial‐centric processes: substrate metabolism, calcium regulation, reactive oxygen species, immune response, and myelination (Depp et al. 2023; Calvo‐Rodriguez et al. 2020). Mitochondrial response to nutrient substrates was recently reported to be impaired in the cortex of heterozygous amyloid precursor protein knock‐in (APP^SAA^) mice (Norambuena et al. 2024). Interestingly, impaired mitochondrial metabolism occurred before the onset of plaques, neuroinflammation, or cognitive decline in APP^SAA^ mice (Norambuena et al. 2024). Therefore, early alterations to the mitochondrial proteome may be a common neurodegenerative proteomic signature within AD model systems. To our knowledge this is the first study to implicate these proteomic pathways (previously observed only in central phenotypes of AD) in peripheral neuronal tissue occurring before overt cognitive impairment, supporting a broader, systemic characteristic of AD‐like pathology. Future studies utilizing advanced imaging techniques or other functional or structural assays following intervention are needed to verify our proteomic findings.

Based on the 4 month age of onset for neuromuscular dysfunction in 5xFAD mice (Brisendine et al. 2024) and the associated sciatic proteome remodeling we defined herein, we hypothesized interventions such as voluntary wheel running or donepezil initiated before onset could delay neuromuscular impairment. While physical activity is recognized as a modifiable risk factor for AD, the effectiveness of exercise to reduce AD risk has been inconsistent. For instance, Yu et al. reported 6 months of cycling training alleviated memory and executive function declines, yet language and attention processing speeds continued to deteriorate. Moreover, the benefits were not sustained over 12 months, as all cognitive measures showed decline (Yu et al. 2021). Others have demonstrated exercise benefits memory when coinciding with improved cardiorespiratory fitness (Morris et al. 2017). Exercise studies using 5xFAD mice have also produced mixed results, some have found that restricting female mice to 3 h of running per day improved cognition, neurogenesis in the hippocampus, and reduced amyloid‐beta accumulation (Choi et al. 2018). In contrast, allowing mice unrestricted access to running wheels did not prevent AD‐like pathology, neuroinflammation, or cognitive decline (Svensson et al. 2020). Our findings revealed that voluntary wheel running attenuated the decline in sciatic CNAP and maintained, but did not increase, nerve‐stimulated muscle torque production. We further demonstrated that exercise maintained the whole sciatic proteome similarly to WT sedentary animals related to mitochondria‐centric processes. These adaptations are consistent with emerging evidence that exercise promotes neuroprotection through mitochondrial remodeling and immune modulation in both central and peripheral tissues (Reddy et al. 2023; Mansoor et al. 2025; O'Reilly et al. 2023). Together, our functional and proteomic findings suggest exercise can attenuate early neuromuscular impairment in a mouse model of AD. However, the disease stage may dictate the effectiveness of exercise as an intervention appears, as we have shown longer term exercise training that extends beyond the onset of neuromuscular dysfunction does not result in mitochondrial adaptations in skeletal muscle (Brisendine et al. 2024). It is possible, therefore, that many human studies of exercise in MCI and AD may occur too late in the disease progression (Sabia et al. 2017), contributing to inconsistent outcomes (Brisendine and Drake 2023).

Analogous to exercise, donepezil treatment prevented declines in nerve‐stimulated muscle torque production and sciatic CNAP, and the proteomic analysis showed distinct enrichment in mitochondrial immune response, calcium buffering, and inflammatory response pathways. Although donepezil is recognized as a treatment for cognitive impairment, its effects on neuromuscular function in AD have yet to be fully elucidated. For instance, some studies have indicated that donepezil treatment can improve skeletal muscle mitochondria respiration, and motor function in individuals with MCI and AD (Rogers et al. 1998; Montero‐Odasso et al. 2015; Morris et al. 2021). In isolated murine skeletal muscle, donepezil treatment prolongs endplate potential and tetanic torque (Redman et al. 2022), suggesting potential benefits beyond merely cognitive symptoms, which is in agreement with our present findings. Donepezil rescues impaired long‐term potentiation in human iPSC‐derived cortical neurons that have been treated with Amyloid‐Beta 42 (Aβ_42_) (Caneus et al. 2024). Donepezil also corrects Aβ_42_‐induced mitochondrial impairment and inflammation in human iPSC systems (Gallo et al. 2024), both proteome pathways through which the protective effect of donepezil, as well as exercise, may be mediated in the present study.

It is important to note that while donepezil treatment begun prior to neuromuscular impairment attenuated decline, donepezil was unable to rescue neuromuscular dysfunction in 5‐month‐old 5xFAD mice once impairment had manifested. This temporal benefit of donepezil's efficacy reflects clinical studies that show declines in physical and cognitive function despite continued treatment (Arai et al. 2018; Courtney et al. 2004). Similar temporal effects have been reported for the NMDA receptor antagonist Memantine in Tg2576 mice (Kodis et al. 2018). As neither donepezil nor exercise had any effect on circulating NfL or hippocampus Aβ oligomers in the current study, the temporal effect of donepezil may be a function of the aggressive pathology in 5xFAD mice. This may explain why longer‐term exercise in 5xFAD did not improve mitochondrial respiration in skeletal muscle (Brisendine et al. 2024). Alternatively, the temporal effect of donepezil may be due to the sciatic proteome remodeling that has already occurred in 5xFAD mice by 5 months of age, thus modifying the pathways needed for donepezil to be effective. However, in human iPSC‐derived cortical neurons treated with Aβ_42_, donepezil‐mediated improvements in mitochondrial health and long‐term potentiation occur without changing elevated p‐Tau(S202/T205) (Gallo et al. 2024). Future studies should further characterize central AD‐like pathology in relation to maintained peripheral neuromuscular function with intervention.

Our study demonstrates both exercise and donepezil treatment resulted in improvements in neuromuscular function in 5xFAD mice, as evidenced by maintained nerve‐stimulated muscle torque production, sciatic nerve CNAP, and proteomic reprofiling. While both interventions modulated shared pathways related to mitochondrial integrity, calcium regulation, and inflammatory response, they also exhibited unique proteomic changes: exercise preferentially enriched pathways associated with the TCA cycle, while donepezil modulated glycogen metabolism and calcium handling pathways. These different yet complementary effects suggest a targeted mechanistic foundation for developing new treatments that incorporate the unique benefits of both exercise and donepezil, or utilizing them simultaneously—offering a potentially synergistic approach to preserve neuromuscular function in AD.

The early manifestation of neuromuscular dysfunction in 5xFAD mice demonstrates peripheral neuromuscular impairments may precede central nervous system AD‐related pathology (Qian et al. 2022; Brisendine et al. 2024; Morris et al. 2021; Norambuena et al. 2024). Our findings support the notion that AD‐like pathology includes peripheral systems through similar pathways identified in AD brains (Depp et al. 2023; Calvo‐Rodriguez et al. 2020). However, our study has several limitations. First, the duration of the interventions was relatively short, which may not fully capture the long‐term effects of voluntary exercise or donepezil treatment or relate to neuroprotective effects of lifelong exercise (Clouston et al. 2013). Second, the study focused on a single mouse model (5xFAD), which does not fully represent the complexity of AD pathology in humans, the complexity of sporadic AD, or the natural aging process. However, recent evidence in fAD iPSC‐derived motor neuron culture systems (Kargazhanov et al. 2026) supports the notion that neuromuscular dysfunction in 5xFAD is relevant for understanding peripheral phenotypes associated with AD. Additionally, we only focused on male mice. We chose to focus on male 5xFAD mice due to our previous characterization of neuromuscular function impairment being more prominent at 4 months of age in the males (Brisendine et al. 2024). The exclusion of female mice does not discount the need to explore the effects of voluntary wheel running and donepezil in female 5xFAD mice, since they too experience early neuromuscular decline (Brisendine et al. 2024) and the increased prevalence of AD in females. Finally, future exercise studies should investigate the interaction between singly housed voluntary running or running paradigms that maintain group housing, such as treadmill training, due to the risk of isolation propelling AD‐like phenotypes. Integrating lifestyle interventions, such as exercise, with pharmacological treatments could potentially offer multifaceted benefits, addressing both cognitive and neuromuscular health in individuals at risk for, or suffering from AD. Advancing our understanding of the interplay between neuromuscular function and AD pathology, both centrally and peripherally, may lead to novel approaches for early identification of risk and potential treatments for AD.

## Author Contributions

Study design: M.H.B., D.Q.N.‐E., C.P.N., C.S.M., and J.C.D. Conducting experiments: M.H.B., D.Q.N.‐E., O.S.W., B.B., J.R.B., D.S.B., S.N.H., J.K.M., and C.P.N. Analyzing data: M.H.B., D.Q.N.‐E., J.K.M., S.P., R.W.G., T.J.J., C.P.N., and J.C.D. Manuscript drafting and review: M.H.B., D.Q.N.‐E., O.S.W., S.P., R.W.G., J.P.T., T.J.J., C.P.N., and J.C.D.

## Funding

This work was supported by an NIH grant (R01AG080731 and K02AG088474) from the National Institute on Aging to J.C.D. NIH‐NIA grant (R00AG070104) from the National Institute of Health was used to support C.P.N. NIH‐NIA grant (R01AG062548) from the National Institute of Health was used to support J.K.M. NIH‐NIA grant (R01AG069781) and NIH‐NIGMS grant (P20GM144269) from the National Institute of Health were used to support J.P.T. and C.S.M.

## Conflicts of Interest

The authors declare no conflicts of interest.

## Supporting information


**Figure S1:** Volcano plots and corresponding KEGG pathway analysis of significant proteins between WT and 5xFAD. (A) Volcano plot of 3‐month‐old WT and 5xFAD sciatic with corresponding KEGG pathway analysis of statistically significant proteins. (B) Volcano plot of 7‐month‐old WT and 5xFAD sciatic with corresponding KEGG pathway analysis of statistically significant proteins.
**Figure S2:** Hindlimb skeletal muscle wet weights following exercise training. (A) Gastrocnemius, (B) Plantaris, (C) Soleus. Data presented as mean ± SEM and two‐way ANOVA was performed. *n* = 6–7 per group.
**Figure S3:** Proteomic changes in the sciatic nerve and STRING network of proteins significantly altered with voluntary wheel running. (A) Left: hierarchical clustering of proteins significantly different between indicated pairings (threshold for clustered proteins was determined by significance between groups). Right: proteins were further grouped using k‐means statistics, breaking the significant genes into 10 protein clusters. (B) Protein–protein interaction networks were generated using STRING analysis (https://string‐db.org) to visualize known and predicted associations among significantly abundant proteins in the sciatic nerve of 5xFAD mice following voluntary wheel running. Each node represents a protein, and edges represent functional associations derived from curated databases, experimental data, text mining, co‐expression, and gene neighborhood. Edge thickness reflects the confidence of the interaction. Functional enrichment and clustering revealed distinct biological modules associated with exercise‐induced proteomic remodeling.
**Figure S4:** Hindlimb skeletal muscle wet weights following donepezil treatment. (A) Gastrocnemius, (B) Plantaris, (C) Soleus. Data presented as mean ± SEM and one‐way ANOVA was performed. *n* = 6–7 per group.
**Figure S5:** STRING network of proteins significantly altered with donepezil treatment. STRING analysis (https://string‐db.org) was performed on proteins significantly abundant in the sciatic nerve of 5xFAD mice treated with donepezil. Nodes correspond to individual proteins, while edges reflect high‐confidence functional interactions based on combined evidence. Functional modules were identified, highlighting pathways responsive to acetylcholinesterase inhibition, including those involved in mitochondrial immune response, inflammation, and calcium handling.
**Figure S6:** Neuromuscular function in 6‐month‐old 5xFAD mice with donepezil treatment: (A) Study design of donepezil treatment intervention. (B) Placebo group tibial nerve stimulated, and direct‐skeletal muscle stimulated muscle function. (C) Tibial nerve‐stimulated skeletal muscle function. (D) Direct muscle stimulated muscle function. (E) In vivo compound nerve action potential (CNAP) of sciatic. *n* = 4 for 5xFAD‐PLCB; *n* = 5 for 5xFAD‐DPNZ. Data presented as mean ± SEM and repeated measures and two‐way ANOVA was performed and Tukey's post hoc test performed when significant interaction between variables was observed (*p < 0.05, **p < 0.01). Panel A created in Biorender.

## Data Availability

The data within this article are available in the article and in its Supporting Information.
