# Supplementary figures and images for: Age‐Dependent Remodeling of the Sciatic Nerve Proteome in 5xFAD Mice Can Be Attenuated by Exercise or Donepezil Treatment to Maintain Neuromuscular Function
Source: Aging Cell. 2026 Jun 16;25(6):e70595. doi: 10.1111/acel.70595 (PMC13270255; doi:10.1111/acel.70595)

Figure S1

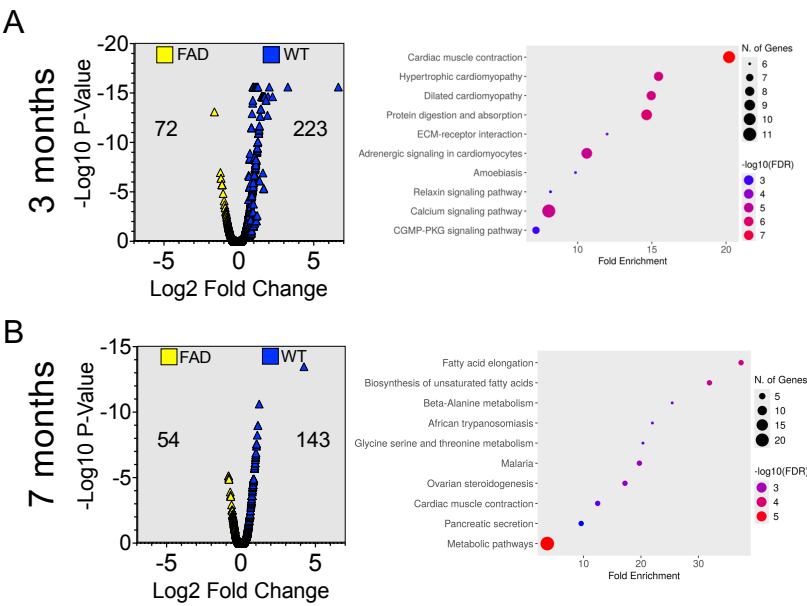

Figure S2

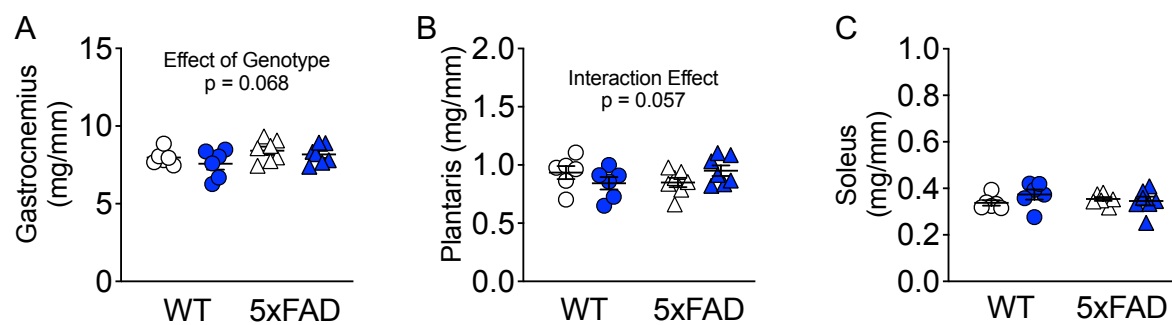

Figure S3

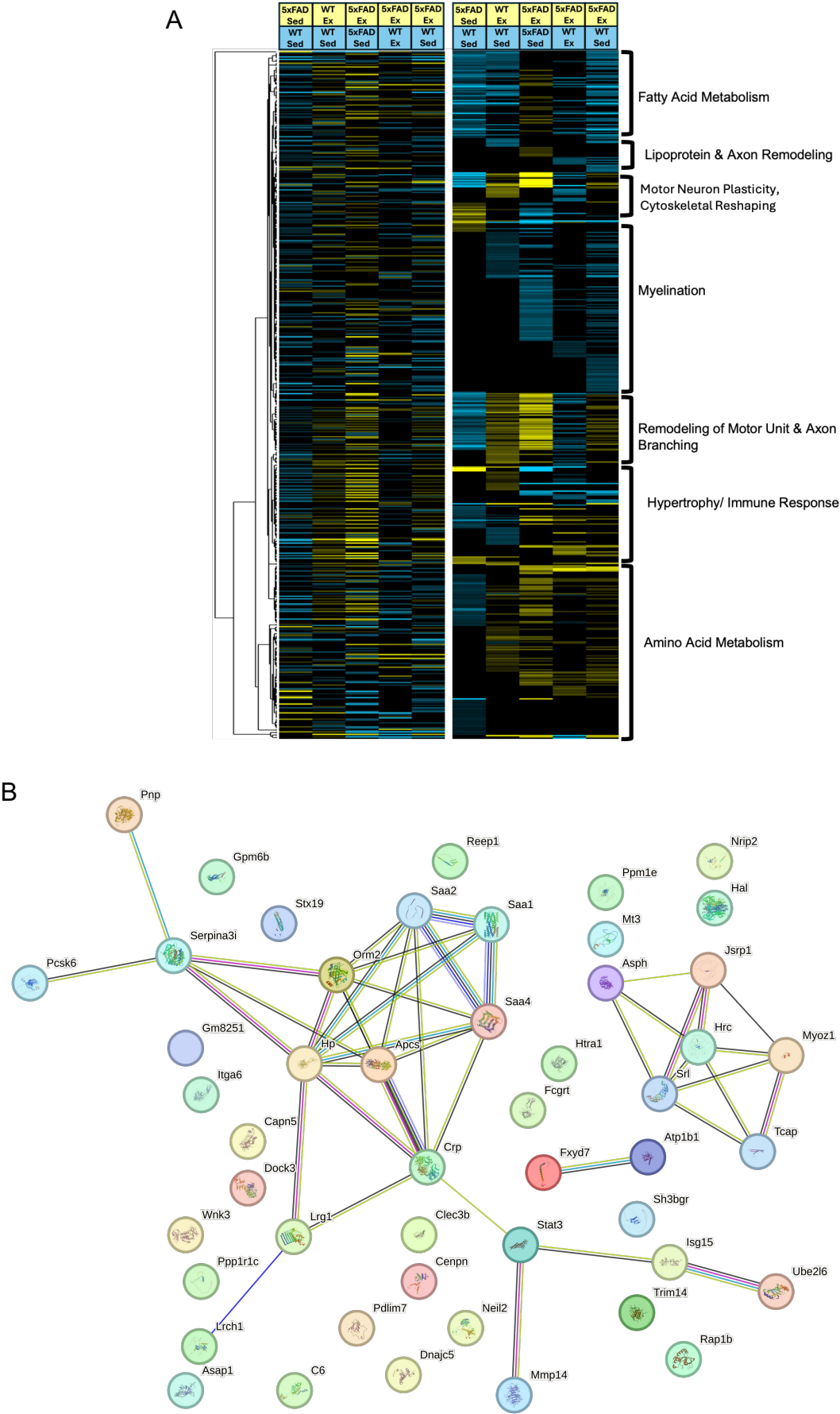

Figure S4

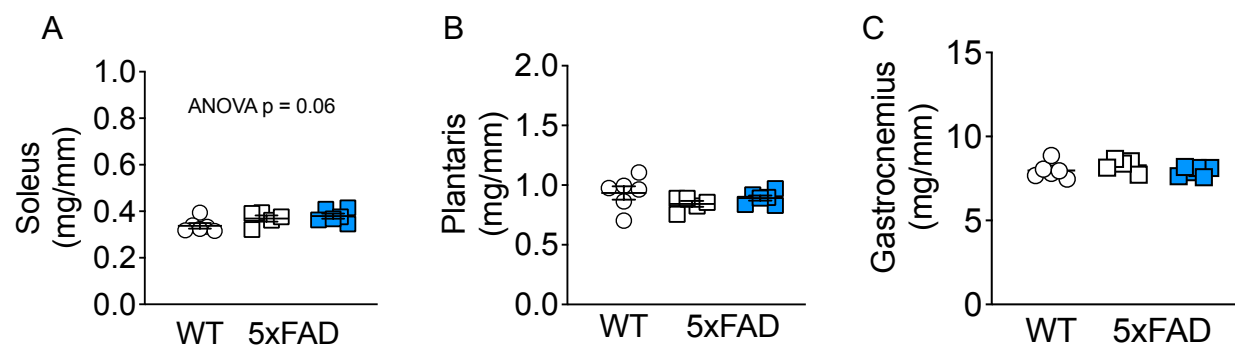

Figure S5

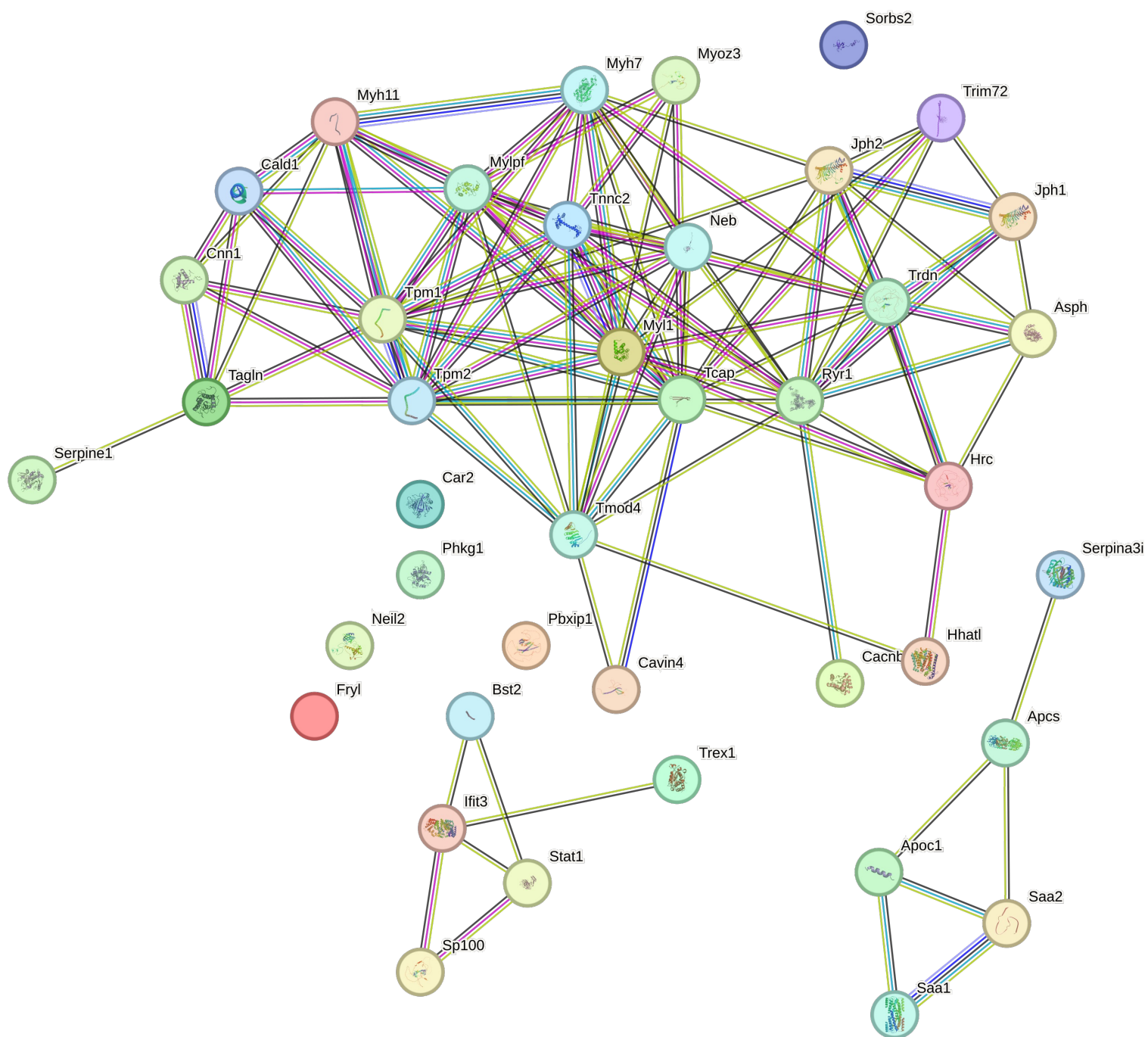

Figure S6

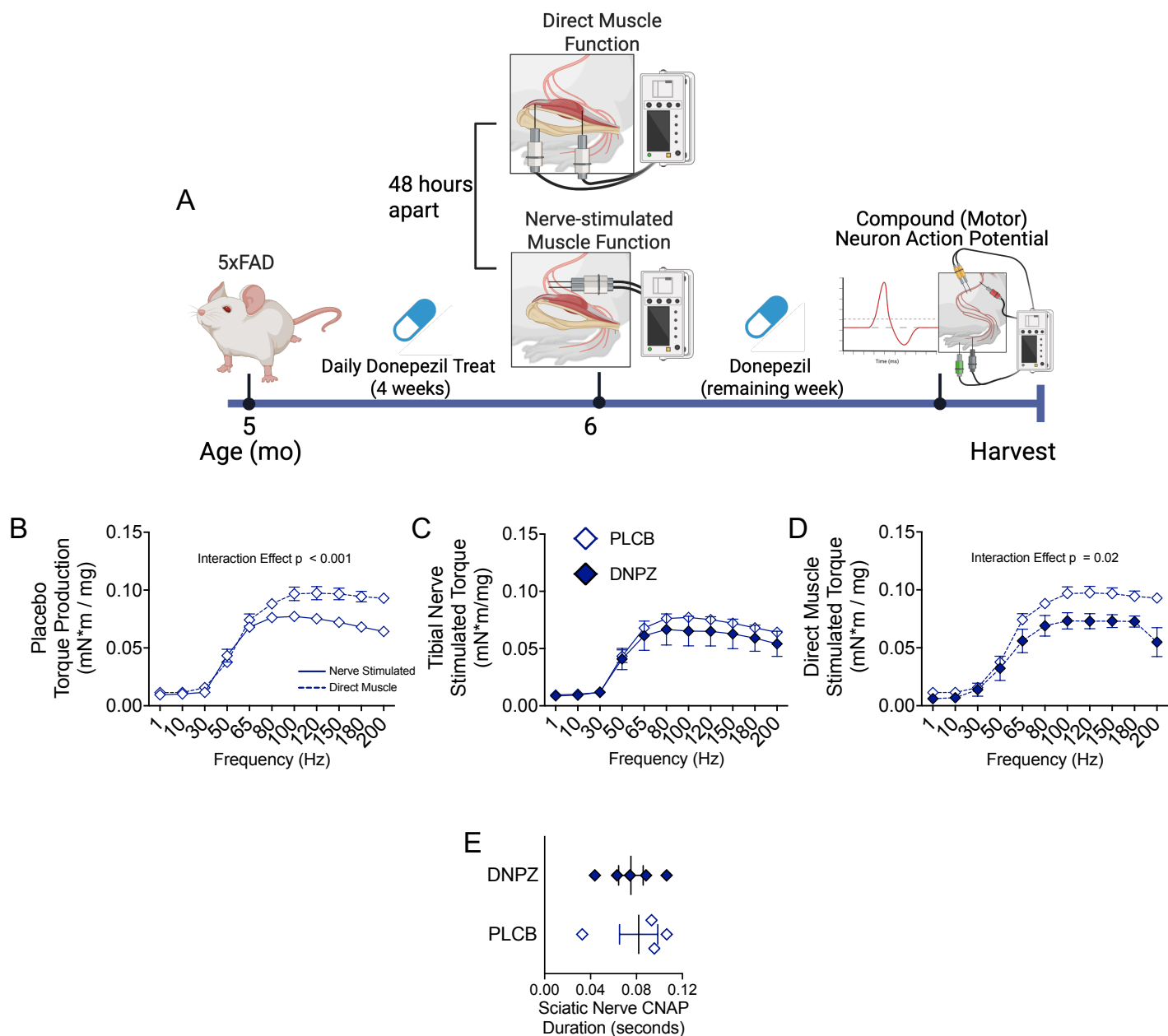

Supplement: Supplementary file 1 — Figure S1: Volcano plots and corresponding KEGG pathway analysis of significant proteins between WT and 5xFAD. (A) Volcano plot of 3‐month‐old WT and 5xFAD sciatic with corresponding KEGG pathway analysis of statistically significant proteins. (B) Volcano plot of 7‐month‐old WT and 5xFAD sciatic with corresponding KEGG pathway analysis of statistically significant proteins. Figure S2: Hindlimb skeletal muscle wet weights following exercise training. (A) Gastrocnemius, (B) Plantaris, (C) Soleus. Data presented as mean ± SEM and two‐way ANOVA was performed. n = 6–7 per group. Figure S3: Proteomic changes in the sciatic nerve and STRING network of proteins significantly altered with voluntary wheel running. (A) Left: hierarchical clustering of proteins significantly different between indicated pairings (threshold for clustered proteins was determined by significance between groups). Right: proteins were further grouped using k‐means statistics, breaking the significant genes into 10 protein clusters. (B) Protein–protein interaction networks were generated using STRING analysis (https://string‐db.org) to visualize known and predicted associations among significantly abundant proteins in the sciatic nerve of 5xFAD mice following voluntary wheel running. Each node represents a protein, and edges represent functional associations derived from curated databases, experimental data, text mining, co‐expression, and gene neighborhood. Edge thickness reflects the confidence of the interaction. Functional enrichment and clustering revealed distinct biological modules associated with exercise‐induced proteomic remodeling. Figure S4: Hindlimb skeletal muscle wet weights following donepezil treatment. (A) Gastrocnemius, (B) Plantaris, (C) Soleus. Data presented as mean ± SEM and one‐way ANOVA was performed. n = 6–7 per group. Figure S5: STRING network of proteins significantly altered with donepezil treatment. STRING analysis (https://string‐db.org) was performed on proteins signifi [file ACEL-25-e70595-s001.pdf]
